# Implementation strategy for advanced practice nursing in primary health care in Latin America and the Caribbean

**DOI:** 10.26633/RPSP.2017.40

**Published:** 2017-04-14

**Authors:** David Oldenburger, Silvia Helena De Bortoli Cassiani, Denise Bryant-Lukosius, Ruta Kristina Valaitis, Andrea Baumann, Joyce Pulcini, Ruth Martin-Misener

**Affiliations:** 1 McMaster University Global Health Office HamiltonOntario Canada McMaster University, Global Health Office, Hamilton, Ontario, Canada.; 2 Pan American Health Organization Pan American Health Organization Washington, D.C. United States of America Pan American Health Organization, Washington, D.C., United States of America.; 3 McMaster University School of Nursing and Canadian Centre for Advanced Practice Nursing Research HamiltonOntario Canada McMaster University, School of Nursing and Canadian Centre for Advanced Practice Nursing Research, Hamilton, Ontario, Canada.; 4 McMaster University World Health Organization Collaborating Centre in Primary Care Nursing and Health Human Resources HamiltonOntario Canada McMaster University, World Health Organization Collaborating Centre in Primary Care Nursing and Health Human Resources, Hamilton, Ontario, Canada.; 5 George Washington University School of Nursing Washington, D.C. United States of America George Washington University, School of Nursing, Washington, D.C., United States of America.; 6 Dalhousie University School of Nursing, Halifax HalifaxNova Scotia Canada Dalhousie University, School of Nursing, Halifax, Nova Scotia, Canada.

**Keywords:** Advanced practice nursing, health policy, legislation, nursing, primary care nursing, primary health care, Latin America, West Indies, Enfermería de práctica avanzada, política de salud, legislación de enfermería, enfermería de atención primaria, atención primaria de salud, América Latina, Indias Occidentales, Prática avançada de enfermagem, política de saúde, legislação de enfermagem, enfermagem de atenção primária, atenção primária à saúde, América Latina, Índias Ocidentais

## Abstract

Advanced practice nursing (APN) is a term used to describe a variety of possible nursing roles operating at an advanced level of practice. Historically, APN roles haves evolved informally, out of the need to improve access to health care services for at-risk and disadvantaged populations and for those living in underserved rural and remote communities. To address health needs, especially ones related to primary health care, nurses acquired additional skills through practice experience, and over time they developed an expanded scope of practice. More recently, APN roles have been developed more formally through the establishment of graduate education programs to meet agreed-upon competencies and standards for practice. The introduction of APN roles is expected to advance primary health care throughout Latin America and the Caribbean, where few such roles exist. The purpose of the paper is to outline an implementation strategy to guide and support the introduction of primary health care APN roles in Latin America and the Caribbean. The strategy includes the adaptation of an existing framework, utilization of recent research evidence, and application of knowledge from experts on APN and primary health care. The strategy consists of nine steps. Each step includes a national perspective that focuses on direct country involvement in health workforce planning and development and on implementation. In addition, each step incorporates an international perspective on encouraging countries that have established APN programs and positions to collaborate in health workforce development with nations without advanced practice nursing.

## SINOPSIS

La enfermería de atención o práctica avanzada es un término que se utiliza para describir una variedad de posibles funciones de enfermería en un nivel más avanzado de ejercicio de la profesión. Históricamente, las funciones del personal de enfermería de práctica avanzada han evolucionado de manera informal debido a la necesidad de mejorar el acceso a los servicios de atención de salud de las poblaciones más desfavorecidas y en riesgo, así como de las personas en las comunidades rurales y remotas que carecen de servicios. Para atender las necesidades de salud, especialmente aquellas relacionadas con la atención primaria, el personal de enfermería fue adquiriendo aptitudes adicionales por medio de la experiencia práctica y, con el transcurso del tiempo, desarrollando un enfoque más amplio del ejercicio de esta profesión. De manera más reciente, las funciones del personal de enfermería de atención avanzada han evolucionado más formalmente mediante el establecimiento de programas de maestría que reúnen las competencias y normas acordadas para el ejercicio de esta especialización. Se prevé que la introducción de las funciones del personal de enfermería de atención avanzada impulsará la atención primaria de salud en América Latina y el Caribe, donde se encuentran pocas funciones similares. La finalidad de este artículo es describir una estrategia de ejecución que guíe y brinde apoyo a la introducción de las funciones del personal de enfermería de atención avanzada en la atención primaria en América Latina y el Caribe. Esta estrategia consiste en la adaptación de un marco existente, la utilización de datos de investigación recientes y la aplicación del conocimiento de expertos en enfermería de atención avanzada y atención primaria de salud. En cada uno de los nueve puntos de los que consta esta estrategia se incluye una perspectiva nacional centrada en la participación directa del país en la planificación y capacitación del personal del área de la salud, así como en la ejecución. Cada punto también contiene una perspectiva internacional sobre cómo alentar a los países que ya han incorporado programas y puestos de enfermería de atención avanzada a colaborar con naciones que aún no cuentan con estas funciones en la capacitación de personal en el área de salud.

## SINOPSE

A enfermagem de práticas avançadas (EPA) é um termo usado para descrever uma variedade de funções possíveis para profissionais de enfermagem atuando em nível avançado de prática. Historicamente, as funções dos EPAs evoluíram informalmente pela necessidade de melhorar o acesso aos serviços de saúde para populações em risco, desfavorecidas, e em comunidades rurais subatendidas e locais remotos. Para atender às necessidades de saúde, principalmente aquelas relacionadas com a atenção primária à saúde (APS), os profissionais de enfermagem adquiriram habilidades adicionais através da experiência prática e, com o passar do tempo, desenvolveram um escopo de prática ampliado. Mais recentemente, as funções dos EPAs foram desenvolvidas de maneira mais formal através do estabelecimento de programas de pós-graduação para proporcionar as competências e padrões de prática acordados. Espera-se que a introdução da enfermagem de práticas avançadas impulsione a APS em toda a América Latina e Caribe, onde poucas destas funções existem. A finalidade deste artigo é traçar as linhas gerais de uma estratégia de implementação para orientar e apoiar a introdução da EPA em atenção primária na América Latina e no Caribe. A estratégia inclui a adaptação de uma estrutura existente, utilização de evidências científicas recentes e aplicação de conhecimento de especialistas a respeito da EPA e da APS. A estratégia consta de nove passos. Cada passo inclui uma perspectiva nacional, centrada na participação direta do país no planejamento e desenvolvimento da força de trabalho de saúde e na implementação. Além disso, cada passo incorpora uma perspectiva internacional para incentivar países nos quais já há programas e funções consagrados de EPA a colaborarem com o desenvolvimento da força de trabalho de saúde em nações onde não há enfermagem de práticas avançadas.

In 2013, the Directing Council of the Pan American Health Organization (PAHO) passed a resolution explicitly stating the need to build on the principles of primary health care (PHC), to promote PHC-based health systems, and to develop training for advanced practice nurses, as part of the strategy for achieving universal health coverage and universal health (1). Comprehensive quality care and equitable access to care for individuals, families, and communities are requisites for universal health. The PAHO Directing Council approved another resolution, in 2014, that outlined key strategies for moving toward universal health (including expanding health services) and that reaffirmed the need for increased, high-quality, more comprehensive human resources for health, in order to meet the unique contextual needs of each country and its population (2).

Advanced practice nursing (APN) is a term used to describe a variety of potential nursing roles (e.g., clinical nurse specialist, nurse practitioner) that operate at an advanced level. An advanced practice nurse is a registered nurse with prior clinical experience who has completed graduate education and developed expertise for an expanded scope of practice as a member of an interdisciplinary team (3, 4). APN roles have existed in North America since the 1960s, and they were initially established to address the need for improved PHC coverage in rural and remote communities. Since then, APN roles have expanded to many other countries and health care settings (5, 6). Today over 70 countries have introduced or are interested in introducing APN roles (7). In the United States of America, there are now over 205 000 advanced practice nurses, the majority of whom work in PHC (8). Research has demonstrated that advanced practice nurses provide efficient, high-quality care; improve patient satisfaction and health outcomes (5, 9); and address PHC concerns related to chronic disease management (10, 11).

In April 2015, PAHO, in conjunction with the School of Nursing and the World Health Organization (WHO) Collaborating Centre in Primary Health Care and Health Human Resources, both at McMaster University, in Hamilton, Ontario, Canada, organized a summit on APN. (WHO Collaborating Centres (CCs) are universities and research institutions with memorandums of understanding to provide research support to WHO and serve as advisors on specific technical areas). Participants at the 2015 summit included nursing leaders from 17 countries of the Americas. Among them were representatives from ministries of health, advisors from PAHO and WHO, deans of schools of nursing, presidents of nursing associations, and nurse researchers. The overall goals of the summit were to define the scope of nursing roles, including those of advanced practice nurses; discuss the context and experiences of countries in the Americas; develop strategies for implementation; and consider the impact that the APN roles could have for improving health (12). A report from the summit outlined strategies for APN role implementation as recommended by the meeting participants (12). Those suggestions provide the basis for the strategy outlined in this paper.

In Latin America and the Caribbean, rural and remote communities and other vulnerable population groups struggle with limited access to basic health services. This health care gap will widen as population sizes increase and chronic diseases becomes more prevalent (1, 13). Expanding comprehensive PHC to address the health needs of underserved populations will require the training of additional high-quality human resources for health (13). Effective implementation of APN roles in PHC in Latin America and the Caribbean could help achieve universal health (1). However, while there are graduate nursing programs in some Latin American and Caribbean countries, most are focused on research or education and not on advanced clinical practice (14).

The purpose of this paper is to present an implementation strategy for enabling the introduction of APN roles. This strategy is designed to provide guidance to countries interested in integrating advanced practice nurses into PHC.

## A NINE-STEP IMPLEMENTATION STRATEGY

**FIGURE 1.**
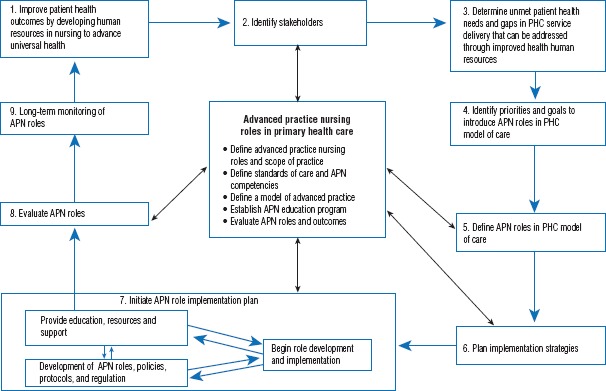
Application of the PEPPA framework to guide advanced practice nursing (APN) implementation in primary health care (PHC) in Latin America and the Carribbean^a^

^a^ Adaptation of the PEPPA framework (15), utilizing objectives developed by the participants at the Advanced Practice Nursing Summit, 15–18 April 2015, McMaster University, Hamilton, Ontario, Canada.

At the April 2015 APN summit, a key recommendation was to ensure the targeted deployment of APN roles by using a systematic strategy to guide countries through the process. To help organize the priorities that the summit participants developed during their meeting, they adapted the “participatory, evidence-based, patient-focused process for advanced practice nurse role development, implementation, and evaluation” (PEPPA) framework (15) (Figure 1). This framework provides a guide to address known barriers to the effective design, implementation, and evaluation of APN roles (9). The framework has been used internationally in at least 16 countries and by various stakeholders to introduce APN roles (16). The framework applies principles of participatory action that are consistent with recommended approaches for health human resource planning (17–19). The adapted framework, with priorities established by summit participants, lays out a systematic implementation strategy.

The implementation strategy (Figure 1) provides a high-level summary that is outlined from two different perspectives: national and international. The national perspective addresses the responsibilities of key stakeholders of the countries that are directly involved in planning, developing, and implementing human resources for health (e.g., national nursing associations, regulatory bodies, policymakers, and national academic institutions). The international perspective focuses on the duties of key stakeholders at the international level that facilitate the implementation process through strategic planning, capacity-building, and promoting collaboration (e.g., PAHO, CCs, universities, and health services researchers).

The strategy includes nine iterative steps. While following the steps sequentially is ideal, use of the strategy is not meant to be prescriptive, and timing of each step can be adapted to individual contexts. The strategy provides the basis to develop the summit’s recommendations into a robust implementation plan.

### Step 1: Improve patient health outcomes by developing human resources in nursing to advance universal health

This step identifies the patient populations to be the focus of activities in subsequent steps. This first step also establishes the scope of the process, from a team, organizational, geographic, or jurisdictional perspective. More specifically, this involves identifying patients and/or populations requiring PHC as the main focus of activities.

The national perspective also addresses the 2015 summit priority to focus APN service delivery on underserved populations (12). This strategy is consistent with other countries where APN roles were introduced to address the needs of underserved populations (5, 6). Moreover, countries have acknowledged that even where there is an adequate number of health care providers per capita for the nation overall, many communities still do not have access to health services because of geographic barriers and poor distribution of providers (1, 2).

### Step 2: Identify stakeholders

At the international level, the priority will be to develop an APN Pan American collaborative network, with the first task of developing a strategy for beginning country-level discussions about the introduction of APN roles.

Summit participants recommended that each country establish a interprofessional task force involving a mix of stakeholders to develop and lead a needs assessment. One common barrier to the effective implementation of APN roles is the lack of understanding and acceptance of the new roles by decisionmakers and health care providers. To address this issue it is important that the task force include medical professionals, allied health professionals, health services managers, and policymakers. Through their engagement, a clear, comprehensive understanding of APN roles being introduced will be developed, and the foundation for creating and accepting new positions to meet agreed-upon priority health needs will be established (5, 12, 20–23).

### Step 3: Determine unmet patient health needs

International stakeholders, such as academic and research institutions, can support national task forces by building the capacity of national stakeholders to conduct needs assessments; identified unmet health needs will inform the priority focus of APN roles.

### Step 4: Identify priorities and goals to introduce advanced practice nursing roles in primary health care

As a final step in the needs assessment, the data collected will be used to determine priorities. This relies on predetermined criteria to identify opportunities that have the greatest likelihood of successfully implementing new APN roles (24). Clearly defined goals are also essential for establishing measurable outcomes to evaluate the effectiveness of APN roles.

International organizations, such as PAHO, can collaborate with countries to identify key health issues and goals. At the national level, countries can use priorityand goal-setting activities to determine outcome-based goals and the best opportunities for utilizing advanced practice nurses in PHC (24).

### Step 5: Define advanced practice nursing roles in primary health care

This step involves identifying strategies and solutions for achieving the goals and expected outcomes identified in Step 4. Each country will be responsible for establishing APN roles and responsibilities that best fit its specific context, by building upon—but limiting overlap with—existing nursing roles.

PAHO, in conjunction with CCs, universities, and other organizations, can provide technical cooperation for the development of a core set of basic competencies. Having similar definitions for APN roles allows for monitoring, evaluation, and comparison of these roles and their effect across Latin American and the Caribbean.

### Step 6: Plan implementation strategies

Stakeholder engagement at the international level will go further, by building a nursing coalition and a narrative to describe the respective roles and contributions of nursing in general and of advanced practice nurses in particular. Nationally, this step is based on four key elements: advocacy, engagement, development, and support.

The successful introduction of APN requires a coalition of nursing leaders and national nursing associations to establish consensus on the types and definitions of advanced practice nurses, and how they will improve outcomes in PHC. Through this consensus, national nursing groups will be able to effectively advocate for APN roles and for the resources to support their introduction into health care systems.

Engaging health services stakeholders is critical for facilitating the acceptance and implementation of APN roles (25). As part of that effort, a meeting of national health leaders from across disciplines and sectors is essential for identifying who will champion these changes and address issues related to APN regulation and scope of practice.

A plan is needed to formalize legislation and create regulations. Experience in other countries has shown that if regulation is not developed in line with the other phases of implementation, the resulting incongruence is a significant barrier for advanced practice nurses working to their optimal scope of practice (26–27). Finally, faculty development is required to establish credible APN education programs. Linkages with faculty in other health professions will create early interprofessional exposure and facilitate greater acceptance of APN roles in the health field (26).

### Step 7: Initiate the advanced practice nursing role implementation plan

The international responsibility is primarily to monitor and evaluate the collaborative efforts among countries, academic institutions, and international stakeholders. PAHO will continue to work with international stakeholders to raise awareness and seek their support in advocating for the development of APN roles and necessary legislation and regulatory policies. Participants at the 2015 summit also asked PAHO to provide technical support for planning and implementing these activities.

In terms of national responsibilities, the seventh step is composed of the following three components:

- Begin role development and implementation. At this stage, an assessment of current graduate nursing programs is needed to identify which universities would be the first to offer an APN education program, and to begin the development of a competency-based curriculum (Step 5 and Step 6). To facilitate this process, PAHO can help universities and organizations that have already promoted or established APN education programs to connect with entities in Latin American and Caribbean countries that want to launch new programs.
- Develop APN roles, policies, protocols, and regulations. This component is heavily reliant on the previous formation of APN coalitions in each country. The priority is for stakeholders to work together to influence governments, universities, and policy-development institutions. Furthermore, engaging health policymakers during the creation of new procedures and guidelines will be important for facilitating implementation of APN roles (26).
- Provide education, resources, and support. Countries in the Americas can develop resources and research capacity with assistance from PAHO, CCs, and universities that have established APN education programs, by continued sharing of resources and research evidence.

### Step 8: Evaluate APN roles

The international responsibility also includes reviewing and evaluating APN roles and implementation frameworks, as well as supporting research to build evidence and to revise the strategy for future application. International stakeholders can also develop and support the use of knowledge translation strategies to inform national health care policymakers and decisionmakers regarding the effective use of APN roles. Nationally, there also needs to be an assessment of the strategy’s overall implementation and impact.

### Step 9: Conduct long-term monitoring of advanced practice nursing roles

The final step is to use the evaluation to identify how APN implementation strategies can be refined to address additional health needs of the population. Implementing APN roles and gaining acceptance for them takes time (25), so countries need to continue monitoring health outcomes in PHC, identify if health needs are being effectively addressed, and assess what further reforms may still be required. Lastly, countries should share their experience of APN implementation with PAHO and with other nations in order to facilitate the implementation process elsewhere.

## DISCUSSION

The development of an implementation strategy is one key step in moving towards implementation of APN roles in Latin America and the Caribbean. The adaptation of the PEPPA framework provides an evidence-based approach for guiding the planning and implementation of APN in PHC. Countries that intend to implement APN roles to advance PHC in their nation can utilize the strategy to develop a systematic plan.

There are various barriers to APN implementation in the Americas. One challenge is the lack of reliable and accurate information in many countries about unmet health needs and gaps in services. A second is the dominance of disease-oriented approaches in health services delivery, which are counter to the patient-centered, health-oriented perspective of comprehensive PHC and APN practice (1, 2, 28). A third obstacle is developing legislation to support expanded scopes of APN practice. A fourth impediment is the limited capacity that academic institutions have for creating APN education programs.

By facilitating ongoing collaboration and supporting capacity-building, PAHO, CCs, and other international stakeholders can assist national stakeholders in addressing these barriers and promoting and implementing adoption of APN roles in PHC. The countries that are best prepared for implementation are those with masters of nursing degree programs that have clinical components, such as Brazil and Chile. With assistance from international academic institutions with established APN programs, masters of nursing degree programs in Latin American and the Caribbean could be adapted to meet core competencies for APN practice in PHC.

Further evidence of the interest in Latin American and the Caribbean comes from the international meetings that have taken place, such as one on establishing core competency models to guide APN curriculum development held in Ann Arbor, Michigan, United States, in April of 2016, as well as meetings with ministries of health to discuss policy-making strategies for effective APN implementation.

### Conclusion

This paper outlined an implementation strategy to help guide implementation of APN roles in Latin America and the Caribbean, based on an existing framework that strategically organizes the key points identified by nursing leaders at the April 2015 summit. Countries can use this strategy as a tool to guide the implementation of APN roles that are relevant to their unique health care needs and system contexts. The ultimate aim is to contribute to the advancement of universal health by strengthening the contribution of nursing through the introduction of advanced practice nursing.

#### Acknowledgments.

Producing this implementation strategy would not have been possible without the development of key priorities by all the participants in the 2015 Advanced Practice Nursing Summit at McMaster University, as well as those persons’ ongoing support and enthusiasm for developing advanced practice nursing in Latin America and the Caribbean.

#### Funding.

No funding was provided to develop this paper.

#### Disclaimer.

Authors hold sole responsibility for the views expressed in the manuscript, which may not necessarily reflect the opinion or policy of the *RPSP/PAJPH* or PAHO.
